# miR-181a-5p targets DDX3X to inhibit the progression of osteoarthritis via NF-ΚB signaling pathway

**DOI:** 10.1186/s13018-023-04073-0

**Published:** 2023-08-16

**Authors:** Peng Zhao, Guobin Ma, Lintong Ma

**Affiliations:** 1 Department of Rheumatology Immunology, 3201 Hospital, 783 Tianhan Avenue, Hantai District, Hanzhong, 723000 China; 2 Department of Hematology, 3201 Hospital, Hanzhong, China

**Keywords:** Osteoarthritis, miR-181a-5p, DEAD-box RNA helicase 3X, Nuclear factor- kappaB

## Abstract

Osteoarthritis (OA) is the most common age-related joint disease, characterized by chronic inflammation, progressive articular cartilage destruction and subchondral osteosclerosis. More and more evidence showed that microRNAs (miRNAs) play a key role in various diseases, but the specific mechanism of miRNAs in OA is not clear. The purpose of this study was to investigate the expression level and role of miR-181a-5p in OA and its related mechanism. Here we identified the key gene DEAD-box RNA helicase 3X (DDX3X) in the OA dataset by bioinformatics analysis. At the same time, miRNAs targeting DDX3X were screened, and miR-181a-5p was selected as the next research object. Then we used different concentrations of interleukin-1 beta (IL-1β)-induced in vitro model of arthritis, and found that IL-1β can stimulate cells to release nitric oxide. The expression levels of miR-181a-5p and DDX3X in mouse chondrocyte cell line ATDC5 induced by IL-1β at a concentration of 10ug/mL were detected by reverse transcription-quantitative polymerase chain reaction (RT-qPCR). IL-1β induced a decrease in the expression of miR-181a-5p and an increase in the expression of DDX3X in ATDC5 cells. mimic miR-181a-5p or inhibitor miR-181a-5p were transfected into ATDC5 cells, and the levels of inflammatory mediators in the cells were detected by enzyme-linked immunosorbent assay, and the results showed that miR-181a-5p could reduce the release of tumor necrosis factor-α, IL-1β, IL-6 and inducible nitric oxide nitric oxide synthase in a cellular model of arthritis. Luciferase reporter assays confirmed that the miR-181a-5p binding site was in the DDX3X gene 3′-untranslated region (3′-UTR), and DDX3X was negatively regulated by miR-181a-5p. Rescue assays confirmed that miR-181a-5p reduced the expression of DDX3X by targeting the 3′-UTR region of DDX3X, thereby reducing the release of inflammatory factors. Finally, in this paper, western blot was used to detect the mechanism of miR-181a-5p regulating OA. The results showed that interfering with the expression of miR-181a-5p could up-regulate the expression of DDX3X protein, increase the expression of nuclear factor- kappaB (NF-κB) related proteins, and reduce the inflammatory response of OA, thereby increasing the secretion of the matrix proteinases MMP-3 and MMP-13. Taken together, the results of the study suggested that miR-181a-5p may be a promising therapeutic target for the treatment of human OA.

## Introduction

Osteoarthritis (OA), also known as degenerative osteoarthritis, is characterized by articular cartilage defects and progressive cartilage degeneration as the main pathological manifestations [1, 2]. It has now become the fourth most common disabling disease in the world [3]. The pathogenesis of OA has not been fully elucidated. At present, the clinical use of symptomatic treatment, conservative treatment and so on, the symptoms of patients can be partially relieved, but the effect is not well [4, 5]. Therefore, finding a better alternative therapy has become an urgent problem to be solved.

microRNAs (miRNAs) are single-stranded endogenously expressed, small non-coding RNAs that express and promote mRNA degradation by inhibiting the translation of messenger RNAs (mRNAs), and are widely found in animals, plants and viruses [6, 7], as well as are associated with musculoskeletal conditions [8–11]. The functional roles of miRNAs in OA have increasingly attracted attention [12]. Studies have shown that the abnormal expression of miR-214-3p in OA activates the nuclear factor-kappaB (NF-κB) signal pathway, which aggravates the development of OA, suggesting that miR-214-3p, as a member of the miRNAs family, may play a role as a regulatory gene in the pathological development of OA and can be used as a new target for the treatment of OA [13, 14]. miR-181a-5p is a member of the miRNAs family and is located on human chromosome 1 [15]. Results showed that IL-1β increased miR-181a-5p and decreased selenocysteine insertion sequence binding protein 2 (SBP2) in a time- and dose-dependent manner, and miR-181a-5p could be controlled by SBP2 in osteoarthritis to reduce antioxidant properties [16]. These studies may indicate that miR-181a-5p plays an important role in osteoarthritis. DEAD-box RNA helicase 3X (DDX3X), is one of the most widely studied and evolutionarily conserved members of the DEAD-box RNA helicase subfamily, and has been reported to participate in several cytosolic steps of mRNA metabolism [17, 18]. DDX3X could regulate the occurrence and development of tumor, virus infection and other diseases, and has the dual role of promoting or inhibiting the occurrence and development of cancer cells. At present, it was considered to be the therapeutic target of cancer and virus.

However, to date, there are limited studies available investigating the effects of miR-181a-5p and DDX3X on OA, and the question of whether miR-181a-5p regulates the expression of DDX3X remains unanswered. Therefore, in the present study, we aimed to examine the effects of miR-181a-5p on OA, as well as the associated mechanisms.

## Materials and methods

### Bioinformation analysis

The Gene expression omnibus (GEO, https://www.ncbi.nlm.nih.gov/gds) online tool [19] was used to screen differential OA datasets: GSE206848, differentially expressed mRNAs in OA were identified using the GEO2R tool. The difference in gene expression was expressed as fold change (FC), and this study set the screening criteria to │logFC│ > 1 and *P* < 0.05. Then, we constructed the protein–protein interactions network (PPI) through online database STRING (Available online: http://string-db.org) [20], and screened the key genes by using huba and Mcode plug-ins in Cytoscape software [21]. Five key genes, PTPN11, FUS, DXXD3 and mcode seed gene SRSF10, CIDEA were obtained. We consulted a large number of related literatures and finally chose DDX3X as the gene for this study. The miRNA of the binding site to DDX3X was predicted by miRNA database (miRmap, Trgetscan, miRDB and miRanda) [22] was used to predict the binding site between miR-181a-5p and DDX3X and then through literature research and miRDB database, it was found that miR-181a-5p and DDX3X had a higher binding score, so miR-181a-5p was selected as the research object to study the mechanism of miR-181a-5p and DDX3X in OA.

### ATDC5 cell line and culture conditions

The mouse chondrocytic cell line ATDC5 was purchased from the American Type Culture Collection (ATCC, Manassas, VA, USA). Cells were maintained at 37 °C with 5% CO_2_, in a 1:1 mixture of DMEM and F12 medium containing 5% (v/v) FBS and 1% penicillin–streptomycin (all from Gibco, Thermo Fisher Scientific), until the culture reached 80% confluence. Then, the recombinant human IL-1β (R&D Systems, Inc.; 0, 10, 100 and 1000 μg /mL) was used to treat the mouse chondrocytic cell line ATDC5 at 37 °C for 12 h to induce cell inflammatory injury.

### Real-time quantitative polymerase chain reaction (RT-qPCR)

Total RNA was extracted with TRIzol™ reagent ((MDBio, Taipei, Taiwan) according to the manufacturer’s instructions from the cultured cells at the concentration of 2 × 10^6^ and the RNA concentration was measured by Nanovue™ spectrophotometer (GE Healthcare). miRNA and mRNA were reverse transcripted with All-in-One™ miRNA first -Strand cDNA Synthesis Kit (GeneCopoeia, USA) and Maxima First Strand cDNA Synthesis Kit (Thermo Fisher Scientific, USA), respectively. Mature miRNA and mRNA were detected by RT-qPCR using Ariamx real time fluorescent quantitative PCR system (Agilent, USA). GAPDH or/and U6 were used as endogenous controls, and the relative expression of miRNA/mRNA were calculated by a 2^−△△ct^ method. The primer sequence is shown in Table 1.

**Table 1:** Primer sequence details

Gene	Sequence (5′- > 3′)
DDX3X	
Forward primer	GTAGCAGTCGTGGACGTTCT
Reverse primer	ACCTGTGTGCCAAGGTTTGA
GAPDH	
Forward primer	AATGGTGAAGGTCGGTGTGA
Reverse primer	CGTGAGTGGAGTCATACTGGAA
U6	
Forward primer	AGAGCCTGTGGTGTCCG
Reverse primer	CATCTTCAAAGCACTTCCCT
miR-181a-5p	
Forward primer	TTAGTGGCTGTCGCAACTTACAA
Reverse primer	CATCTTCAAAGCACTTCCCT

### Enzyme-Linked Immunosorbent Assay (ELISA)

To measure the concentration of inflammatory cytokines in the conditioned medium, ELISA was measured using the method described in our previous work [23]. According to the manufacturer’s procedure, the mouse ELISA kit (Aviva Systems Biology, OKEH00026) was used to determine the interleukin 1β (IL-1β), interleukin 6(IL-6), tumour necrosis factor alpha (TNF-α) and inducible nitric oxide synthase (iNOS) produced by the mouse chondrocytic cell line ATDC5 in the conditioned medium.

### Luciferase reporter assays

The luciferase assays were carried out using the Dual-luciferase Reporter Assay System (Promega, Madison, WI, USA). Briefly, HEK-293 T cells were co-transfected with mimic miR-181a-5p or miR NC and psicheck2-reporter luciferase vector containing a specific sequence of wild-type or mutant DDX3X fragment, using siRNA transfection (Invitrogen, NY, USA). Cells were collected and lysed for luciferase detection 48 h after transfection. The relative luciferase activity was normalized against to the Renilla luciferase activity.

### Western blot assays

The ATDC5 cells at the concentration of approximately 3 × 10^5^ were seeded in 6-well plates. The cell density was reached 80% before transfected with plasmids. 48 h after transfection, cells cultured medium was removed and RIPA was added to extract protein. The concentration of protein was measured by BCA protein quantification assay kit (Beyotime, Shanghai). Then proteins samples were separated by SDS-PAGE (5% of concentration gel and 12% of separation gel) and then transferred to PVDF membranes. The membranes were blocked by 5% skimmed milk for 1.5 h and incubated with primary antibodies [DDX3X, P65, p-P65, IkappaB (IkB-α), p-IkappaB (p-IkB-α), MMP-3, MMP-13]at 1:1000 dilution overnight at 4 °C. After washing the bands for three times with TBST, the secondary antibody of goat anti-rabbit was used to incubated for 1 h at 1:10,000 dilution at room temperature. According to the manufacturer’s protocol, the protein membranes were detected with ECL immunoblotting kit (Millipore, USA).

### Statistical analyses

The data are expressed as the mean ± standard deviation. All experiments were repeated at least three times. Comparisons among values for more groups were performed by one-way analysis of variance. Holm’s test was applied for analysis of differences between two different groups. *P* < 0.05 was considered to indicate a statistically significant difference.

## Results

### Screening of significant differentially expressed genes in OA

**Fig. 1:**
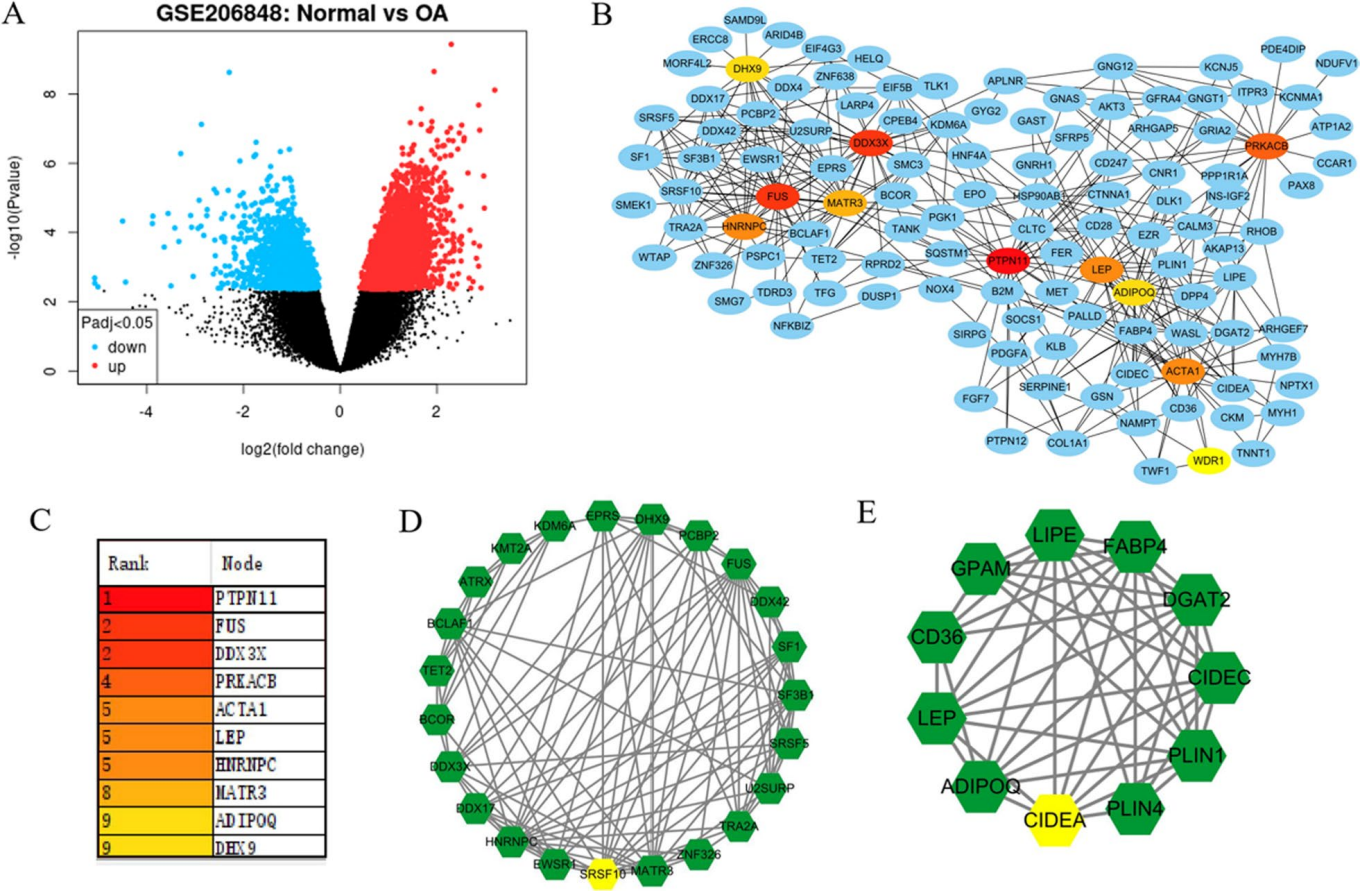
Screening of differentially expressed genes in OA. **A** Identification of differentially expressed genes from GSE2066848 dataset. **B** The PPI network is established through STRING database. **C** Some hub genes were identified by node degree. **D** and **E** the Mcode plug-in is emplyed to screen key genes. Abbreviation: PPI: protein–protein interactions network; OA: osteoarthritis

**Fig. 2:**
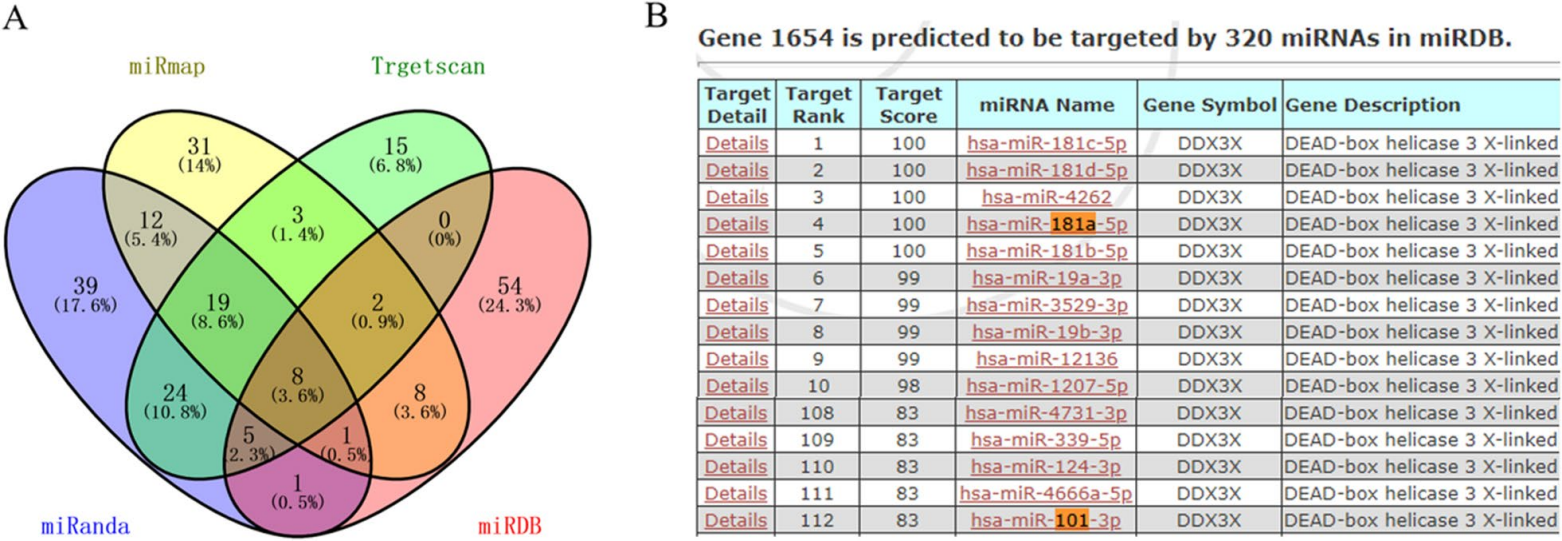
Identification of key genes. **A** Identification of 8 miRNA genes targeting DDX3X from the expression profile databases miRmap, Trgetscan, miRDB and miRanda. **B** miRNAs with better targeting binding to DDX3X predicted by miRDB database. Abbreviation: DDX3X: depletion of DEAD-box RNA helicase 3X

As showed in Fig. 1A, the GSE206848 dataset was used to identify differentially expressed genes in OA. Using the STRING online database to establish PPI network of differentially expressed genes (Fig. 1B). According to node degree, we identified some hub genes among these significant DEGs (Fig. 2C). In order to better visualize, the interactions of some differentially expressed central genes were reconstructed using Mcode plug-in software (Fig. 1D, E).

### Identification of key genes

We extracted 84, 76, 79 and 109 differentially expressed genes from the expression profile databases miRmap, Trgetscan, miRDB and miRanda, respectively. After integrated bioinformatical analysis, a total of 8 miRNA genes targeting DDX3X were identified from the four profile databases (Fig. 2A) (*P* < 0.05), including hsa-miR-101-3p; hsa-miR-181a-5p; hsa-miR-181b-5p; hsa-miR-181c-5p; hsa-miR-124-3p; hsa-miR-382-5p; hsa-miR-495-3p and hsa-miR-181d-5p. Then through a large number of literature research, we found that miR-181a and miR-101-3p are closely related to OA. Through the identification of miRDB database and the combination of miRNAs with DDX3X targeting, it is found that miR-181a-5p and DDX3X have better targeting binding force (Fig. 2B) (*P* < 0.05), so miR-181a-5p was selected as the follow-up research object.

### *IL-1β induces inflammatory injury of chondrocytes *in vitro* and regulates the expression of miR-181a-5p and DDX3X*

**Fig. 3:**
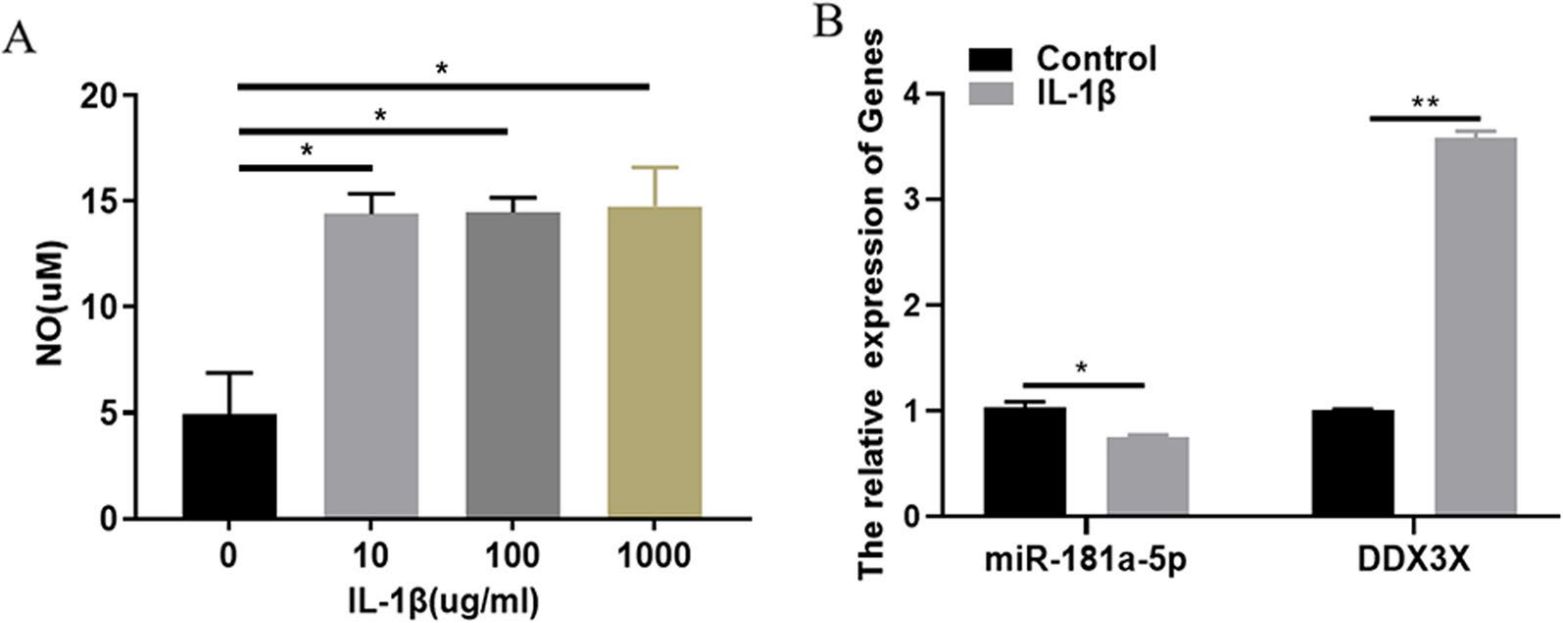
IL-1 β induces inflammatory injury of chondrocytes in vitro and regulates the expression of miR-181a-5p and DDX3X. **A** IL-1β-treated chondrocytes stimulated the cells to release NO in a concentration-dependent manner. **B** After chondrocytes were treated with IL-1β, the expression level of miR-181a-5p decreased and the expression level of DDX3X increased. ^ns^*P* > 0.05; ^*^P < 0.05; ^**^P < 0.01. DDX3X: depletion of DEAD-box RNA helicase 3X; IL-1β: interleukin 1β

To determine whether chondrocytes affected miR-181a-5p and DDX3X gene expression after IL-1β treatment, we treated chondrocytes with different concentrations of IL-1β. We observed that the concentration of nitric oxide increased after the chondrocytes were treated with IL-1β, but there was no significant difference in the concentration of nitric oxide released by the cells after the chondrocytes were treated with different concentrations (10, 100 and 1000 μg/mL) of IL-1β (Fig. 3A) (*P* < 0.05). Therefore, the in vitro articular chondrocyte model of arthritis was established by treating chondrocytes with 10 μg/mL concentration of IL-1β. Then in order to observe the relationship between osteoarthritis and miR-181a-5p, DDX3X expression, we treated chondrocytes with 10 ug/mL concentration of IL-1β and found that IL-1β could stimulate chondrocytes to down-regulate the expression of miR-181a-5p and up-regulate the expression of DDX3X (Fig. 3B) (*P* < 0.05). The results suggested that the occurrence of osteoarthritis is related to the abnormal expression of miR-181a-5p and DDX3X.

### miR-181a-5p reduces inflammatory cytokine release in a cellular model of arthritis induced by IL-1β

**Fig. 4:**
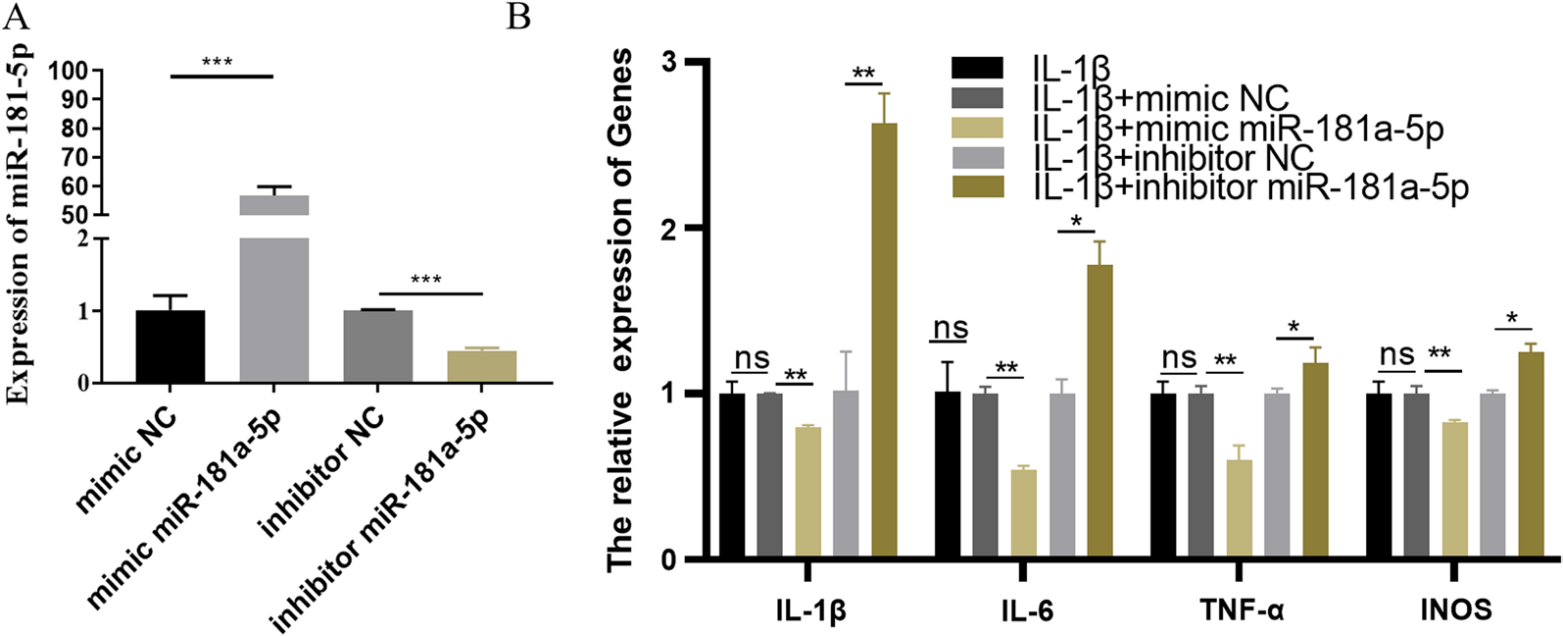
miR-181a-5p reduces inflammatory cytokine release in a cellular model of arthritis induced by IL-1β. **A** RT-qPCR detection of the transfection efficiency of miR-181-5p in a cellular model of arthritis. **B** miR-181a-5p could reduce the release of inflammatory factors (IL-1β, IL-6, TNF-α and iNOS) from ATDC5 cells induced by IL-1 β. ^ns^*P* > 0.05;^*^ *P* < 0.05; ^**^ *P* < 0.01; ^***^ *P* < 0.001. IL-1β: interleukin 1β; IL-6: interleukin 6; TNF-α: tumour necrosis factor alpha; iNOS: inducible nitric oxide synthase

To further investigate the role of miR-181-5p in OA, ATDC5 cells were transfected with mimic miR-181a-5p or inhibitor miR-181a-5p for 24 h, and the treated with 10 μg/mL IL-1β for another 24 h. The transfection efficiency of miR-181-5p in ATDC5 cells was first detected by RT-qPCR, so that the follow-up experiment could be carried out smoothly. As shown in Fig. 4A, the level of miR-181a-5p mRNA increased significantly in ATDC5 cells transfected mimic miR-181a-5p, while the level of miR-181a-5p mRNA decreased significantly after transfection of inhibitor miR-181a-5p (*P* < 0.05). The results suggested that the transfection efficiency of miR-181a-5p in ATDC5 cells is in line with the expectation and could be used in follow-up experiments. The abnormal expression of miR-181a-5p affects the release of proinflammatory cytokines and contributes to the further analysis of the clinical symptoms of OA. Subsequently, the cytokines in chondrocytes overexpressing miR-181a-5p or interfering with miR-181a-5p after IL-1β stimulation were measured by ELISA. The upregulation of miR-181a-5p suppressed the IL-1β-induced expression of pro‑inflammatory cytokines, compared with chondrocytes transfected with the mimic NC (Fig. 4B) (*P* < 0.05). When IL-1β-induced chondrocytes with transfected inhibitor miR-181a-5p, the level of inflammatory cytokines showed the opposite result (Fig. 4B) (*P* < 0.05). These results suggested that miR-181a-5p protects chondrocytes from IL-1β-induced cellular inflammation.

### DDX3X gene has a miR-181a-5p binding site and is negatively regulated by miR-181a-5p

**Fig. 5:**
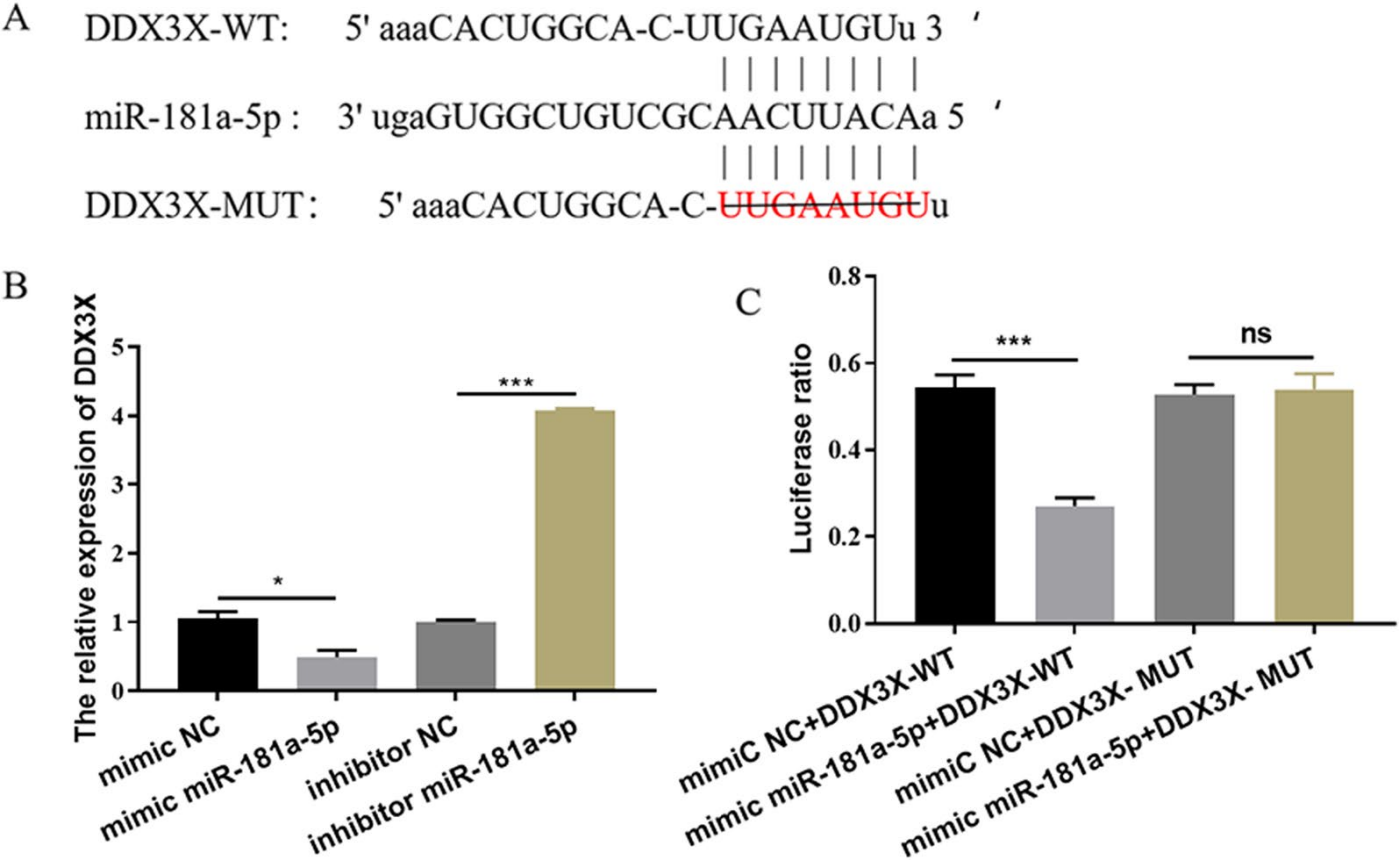
DDX3X gene has a miR-181a-5p binding site and is negatively regulated by miR-181a-5p. **A** Bioinformatics analysis predicted that DDX3X is the target gene of miR-181a-5p. **B** Dual -luciferase reporter gene assay was used to detect the luciferase activity after co-transfected with miR-181a-5p mimic and DDX3X -WT/MUT. **C** The relative expression of DDX3X regulated by miR-181a-5p. ^ns^*P* > 0.05; ^***^ *P* < 0.001. DDX3X: depletion of DEAD-box RNA helicase 3X

In order to predict the target genes of miR-181a-5p, the prediction was made through the bioinformatics database and verified by the double luciferase experiment report. The results revealed that a binding site for miR-181a-5p was in the DDX3X mRNA 3′-UTR (Fig. 5A). In order to confirm that miR-181a-5p binds to DDX3X mRNA 3′-UTR region and leads to translational repression, we carried out double luciferase gene reporting experiment to detect the relative luciferase activity of cells. The data showed that transfection of mimic miR-181a-5p significantly decreased the activity of relative luciferase in mouse chondrocytic cell line ATDC5 transfected with DDX3X-WT, but did not affect the activity of relative luciferase in cellls transfected with DDX3X-MUT (Fig. 4B) (*P* < 0.05), suggesting that there was a targeted regulation relationship between miR-181a-5p and DDX3X mRNA. We further detected the changes of DDX3X mRNA level in mouse chondrocyte cell line ATDC5 transfected with mimic miR-181a-5p or inhibitor miR-181a-5p by RT-qPCR assays. As showed in Fig. 5C, the level of DDX3X mRNA in mimic miR-181a-5p group decreased significantly, but it showed the opposite result in inhibitor miR-181a-5p group (*P* < 0.05). These results showed that miR-181a-5p could target DDX3X and regulate its mRNA level changes.

miR-181a-5p targets to regulate the 3′ UTR of DDX3X to reduce the expression of DDX3X, thereby reducing the release of inflammatory cytokines in arthritis model cells.

**Fig. 6:**
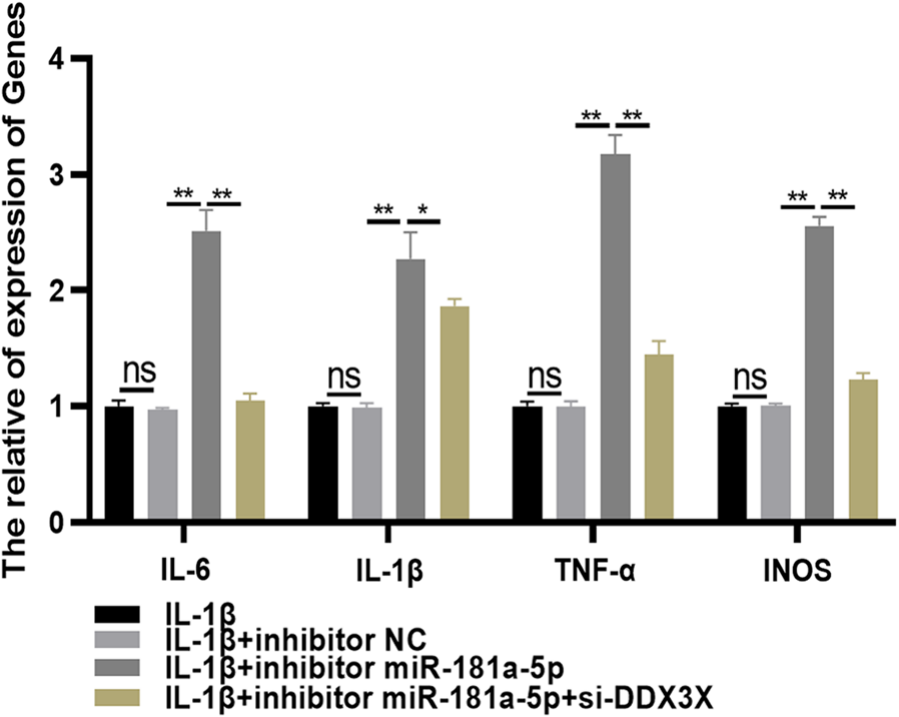
miR-181a-5p targets to regulate the 3′UTR of DDX3X to reduce the expression of DDX3X, thereby reducing the release of inflammatory cytokines in arthritis model cells. ^ns^*P* > 0.05;^*^ *P* < 0.05; ^**^ *P* < 0.01. TNF-α: tumor necrosis factor-α; IL-6: interleukin-6; IL-1β: interleukin-1β; INOS: inducible nitric oxide synthase

In order to understand whether miR-181a-5p can target DDX3X gene expression, thereby affecting the level of inflammatory factors in cellular model of arthritis, rescue assays were performed in this paper. As shown in Fig. 6, there was no significant difference in IL-6, IL-1β, TNF-α and iNOS levels between IL-1β group and IL-1β + inhibitor NC group. Compared with IL-1β + inhibitor NC group, the IL-6, IL-1β, TNF-α and iNOS levels were significantly increased in IL-1β + inhibitor miR-181a-5p group (*P* < 0.05). Compared with the IL-1β + inhibitor miR-181a-5p group, the D IL-6, IL-1β, TNF-α and iNOS levels in the IL-1β + inhibitor miR-181a-5p + si-DDX3X group were significantly decreased (*P* < 0.05). The results of this study showed that miR-181a-5p could target DDX3X to reduce the production of inflammatory factors in chondrocyte cell line ATDC5 induced by IL-1β.

### Interfering with the level of miR-181-5p gene can up-regulate the expression of DDX3X and regulate the level of NF-κB-related proteins

**Fig. 7:**
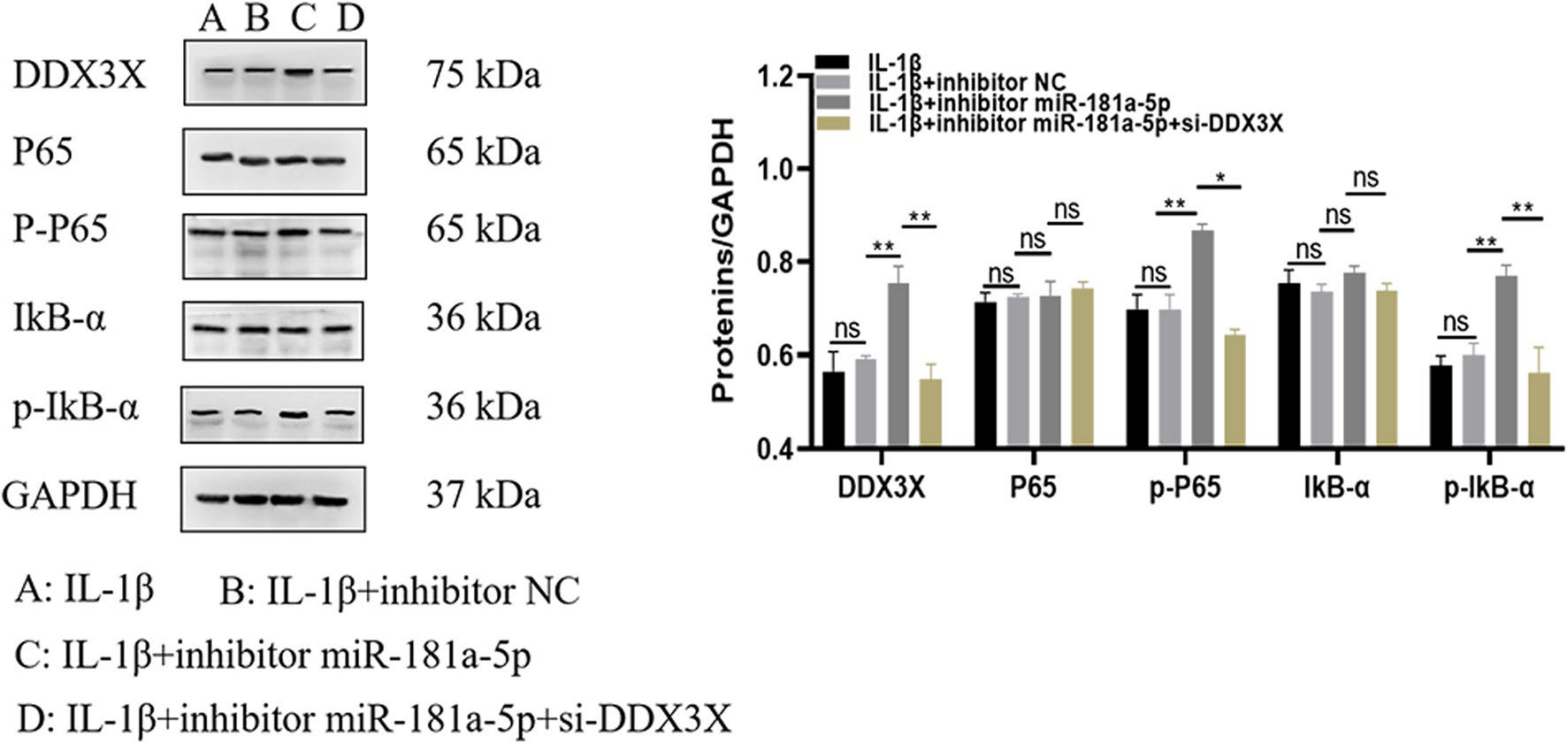
Interfering with the level of miR-181-5p gene can up-regulate the expression of DDX3X and regulate the level of NF-ΚB-related proteins. ^ns^*P* > 0.05;^*^ *P* < 0.05; ^**^ *P* < 0.01. NF-κB: nuclear factor-kappa B; DDX3X: depletion of DEAD-box RNA helicase 3X

In order to investigate the specific mechanism of the effect of DDX3X gene regulated by miR-181-5p on osteoarthritis, chondrocytes in inhibitor NC, inhibitor miR-181a-5p and inhibitor miR-181a-5p + si-DDX3X groups were stimulated with 10 μg/mL of IL-1β, and the cells were collected to detect the expression of proteins related to NF-κB signal pathway. As shown in Fig. 7, there was no significant difference in DDX3X, p-P65 and p-IKB-α protein levels between IL-1β group and IL-1β + inhibitor-5p NC group. Compared with IL-1β + inhibitor NC group, DDX3X, p-P65 and p-IKB-α protein levels were significantly increased in IL-1β + inhibitor miR-181a-5p group (*P* < 0.05). Compared with the IL-1β + inhibitor miR-181a-5p group, the DDX3X, p-P65 and p-IKB-α protein levels in the IL-1β + inhibitor miR-181a-5p + si-DDX3X group were significantly decreased (*P* < 0.05). The results of this study showed that interfering with the expression of miR-181a-5p could up-regulate the expression of DDX3X gene and increase the expression of NF-κB proteins such as p-P65 and p-IKB-a.

### Interfering with the levels of the miR-181-5p gene can inhibit the catabolic activity in arthritis model cells

**Fig. 8:**
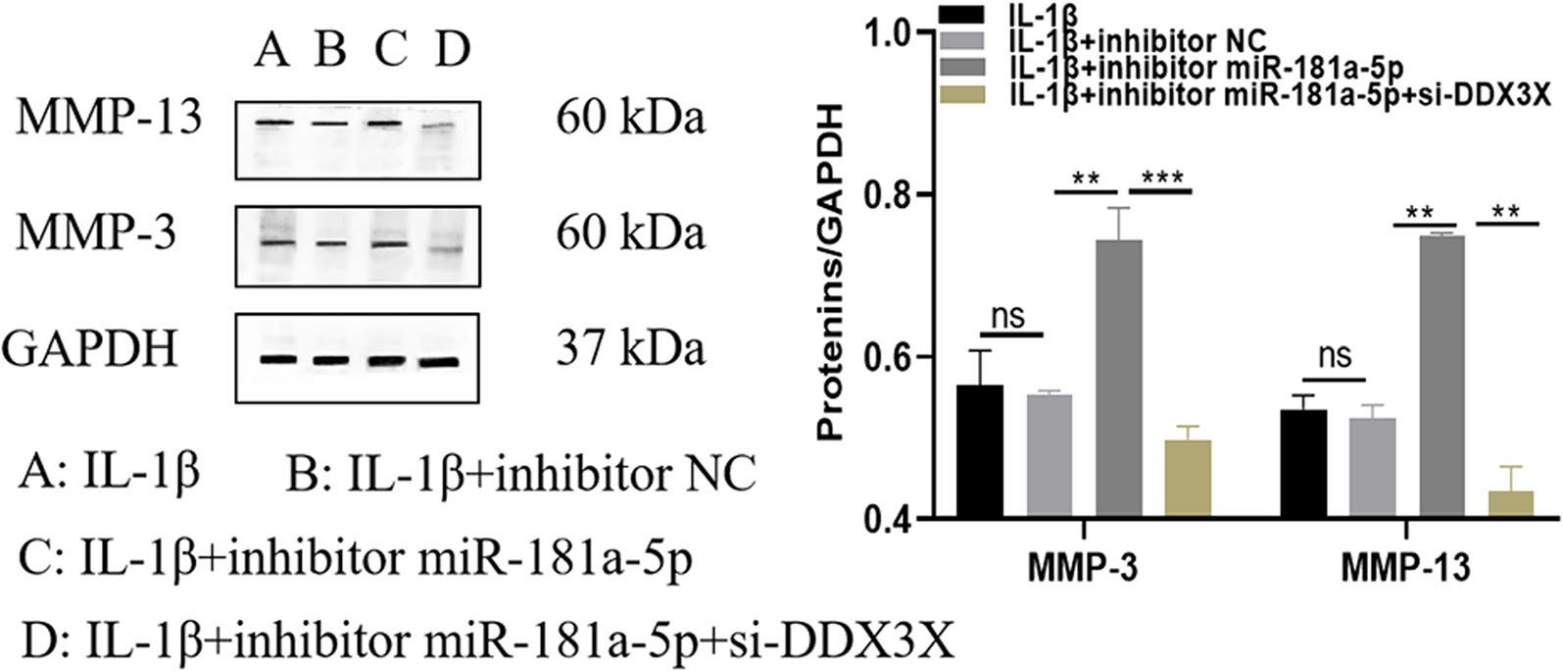
Interfering with the levels of the miR-181-5p gene can inhibit the catabolic activity in arthritis model cells. ^ns^ *P* > 0.05; ^*^ *P* < 0.05; ^**^ *P* < 0.01. MMP-3: Matrix metalloproteinase-3; MMP-13: Matrix metalloproteinase-13

To verify the relationship between chondrocyte activity and the pathogenesis of OA, chondrocytes in inhibitor NC, inhibitor miR-181a-5p and inhibitor miR-181a-5p + si-DDX3X groups were stimulated with 10 μg/mL of IL-1β, and the cells were collected to detect the proteins expression of MMP-3 and MMP-13. As shown in Fig. 8, there was no significant difference in MMP-3 and MMP-13 protein levels between IL-1β group and IL-1β + inhibitor-181-5p NC group. Compared with IL-1β + inhibitor NC group, MMP-3 and MMP-13 protein levels were significantly increased in IL-1β + inhibitor miR-181a-5p group (P < 0.05). Compared with the IL-1β + inhibitor miR-181a-5p group, the MMP-3 and MMP-13 protein levels in the IL-1β + inhibitor miR-181a-5p + si-DDX3X group were significantly decreased (P < 0.05). This result suggests that the development of OA is not only related to NF-κB signal pathway, but also possibly to the catabolic activity of chondrocytes, interfering with the expression of miR-181a-5p promoting the secretion of cartilage matrix degrading enzymes.

## Discussion

OA was a chronic and degenerative disease characterized by joint pain and swelling [24]. With the change of the course of the disease, it can lead to joint deformity or even disability [25]. OA was the most common disease in arthritis, and it has a high incidence in middle-aged and elderly people. Inflammatory mediators can reduce the self-repair ability of chondrocytes and damage the normal function of joints [26]. The inhibition of inflammatory response is of great value in the treatment of OA. Clinical treatment of OA, mainly through the joint hyperplasia of synovium, exfoliated cartilage and other removal, and then reduce the secretion of inflammatory factors in the joint to achieve the purpose of relieving knee joint pain [27]. Thus, understanding the release of inflammatory mediators and the molecular mechanism of OA is of great significance for the treatment of OA.

In this paper, first, we screened the osteoarthritis dataset (GSE206848) by GEO for differential gene analysis, and then used Cytoscape and Mcode plugin to screen out the key gene DDX3X. Inflammation is the main pathological response in the pathogenesis of OA, which could cause chondrocyte apoptosis, extracellular matrix degradation and bone hyperplasia, damage cartilage tissue, and play an important role in the progression of OA [28, 29]. According to the literature review, DDX3X can regulate the physiological process of cellular inflammation by promoting the activation of NLRP3 inflammasome [30]. Based on this, it is speculated that DDX3X may affect the inflammatory response of OA and may have an important impact on the occurrence of OA. Through bioinformatics data and literature research, we found that miR-181a-5p and DDX3X have a good targeting binding force, so we choose miR-181a-5p as the in-depth research object of this paper.

To explore whether miR-181a-5p and DDX3X are involved in OA pathogenesis, we established an IL-1β model using mouse chondrocyte cell line ATDC5. The results showed that IL-1β decreased miR-181a-5p and increased DDX3X. Thus, we hypothesized that miR-181a-5p may exert protective effects against OA through the negative regulation of the expression of DDX3X. In order to prove this hypothesis, we used mimics sequence and inhibitor sequence to up- or down-regulate the expression of miR-181a-5p in ATDC5 cells, and carried out a series of studies. In this study, ELISA was used to detect the level of inflammation in ATDC5 cells induced by IL-1β. The results showed that miR-181a-5p could reduce the release of IL-1β, IL-6, TNF-α and iNOS inflammatory factors in cellular model of arthritis. Inflammatory factors play an important role in promoting the progression of OA, especially IL-1β and IL-6 play a key role in the pathological process of OA [31, 32]. IL-1β and IL-6 could stimulate the production of pain factors such as NO, iNOS and prostaglandin E2 (PGE2) [31, 33, 34]. These pain factors were highly expressed in OA and were significantly related to the progression of OA. TNF-α was another inflammatory cytokine that leads to the pathogenesis of OA [35, 36]. Studies have shown that the content of TNF-α is significantly higher in OA [37]. This finding suggested that miR-181a-5p could inhibit the release of inflammatory cytokines from osteoarthritis cells.

In order to further understand the possible mechanism of miR-181a-5p regulating the inflammatory response of osteoarthritis cells, we found that DDX3X is the target of miR-181a-5p through bioinformatics analysis. The double luciferase report assays confirmed this prediction and proved that DDX3X was directly targeted by miR-181a-5p at its 3′-UTR. In addition, we also examined the expression level of DDX3X when the level of miR-181a-5p was altered. By examining the overexpression (by transfection with mimic miR-181a-5p) or downregulation (by transfection with inhibitor miR-181a-5p) of the level of miR-181a-5p, we found that the expression level of DDX3X negatively correlated with miR-181a-5p. As an important epigenetic regulatory factor, miRNA exists widely in the biological world. miRNA is a small non-coding RNA with a length of 18–25 nucleotides, which is highly conserved [38, 39]. It mainly binds to the 3′-UTR of the target gene mRNA, resulting in mRNA degradation or translation inhibition, and plays an apparent modification role at the post-transcriptional level in various stages of cell differentiation, development, proliferation, apoptosis and so on [40]. The results of this study confirmed that miR-181a-5p can specifically regulate the mRNA level of DDX3X gene, and then affect the development of OA. At the same time, rescue assay further confirmed the targeting effect of miR-181a-5p and DDX3X, regulating the changes of inflammatory cytokines (IL-1β, IL-6, TNF- α and iNOS) in OA cells, and then aggravating the development of the disease.

NF-κB was a multidirectional transcription factor that widely exists in eukaryotic cells [41]. It participates in a variety of biological processes and regulates the expression of a variety of cytokines, biochemical factors and enzymes [42]. Many studies have shown that NF-κB signal pathway is one of the main pathways leading to cartilage catabolism in the course of OA and plays a key role in the progression of OA [43, 44]. Upon stimulation by IL-1β, NF-κB detaches from IκB and translocates into the nucleus to regulate inflammatory cytokine expression, which induces destruction of the articular joint, leading to the onset and progression of OA. A previous study showed that the TLR4/NF-κB signaling pathway is a vital mechanism for the regulation of inflammatory responses in human OA chondrocytes [45]. In this study, it was shown that interfering with miR-181a-5p expression, targeted to increase the expression of DDX3X, increased the expression of NF-κB-related proteins, and alleviated OA inflammation. These results suggested that miR-181a-5p inhibited the IL-1β‑induced inflammatory response by inhibiting the NF-κB signaling pathway.

MMPs are zinc-dependent endopeptidases, also known as matrix proteins. MMPs are a major group of enzymes that regulate cell matrix composition and can degrade all components of the extracellular matrix. Previous study [46] have shown that, a range of MMPs, including MMP-13, MMP-2, MMP-9, and MMP-3, play a key role in OA cartilage destruction through the degradation of aggrecans and collagen. Where the MMP-13 is the enzyme responsible for the degeneration of the cartilage extracellular matrix and the degenerative process of OA pathogenesis, has a rate-limiting role in collagen degradation. Therefore, the increased activity of MMP-13 plays an important role in the induction and pathogenesis of OA. Other MMPs, such as MMP-2, MMP-3 and MMP-9 were also elevated in OA. These enzymes degrade the non-collagenous matrix components of the joint. In addition, study has shown that cytokines such as IL-1β and TNF-α stimulate the expression of matrix metalloproteinases [47]. In this study, interference with the expression of miR-181a-5p increased expression of NF-KB associated cytokines and increased expression levels of MMP-3 and MMP-13 proteins. These results suggest that interference with miR-181a-5p may lead to the development of OA by increasing inflammatory cytokines and DDX3X expression and subsequently inducing increased MMP-3 and MMP-13 activities.

Taken together, the present study demonstrated that miR-181-5p inhibits IL-1β- induced occurrence of OA by negatively regulating the expression of DDX3X gene, which may achieve by inhibiting the NF-κB pathway and secretion of the matrix proteinases. The miR-181a-5p/DDX3X/NF-κB axis may be a promising target for the treatment of OA. Although this study has important implications for elucidating the mechanism of OA, there are still shortcomings in this study. Due to limited clinical sample resources, the expression verification of miR-181a-5p and DDX3X in clinical samples is lacking. In the future, we will continue to collect samples for further research.

## Data Availability

Data supporting the findings of this study are available from the corresponding author upon reasonable request.

## References

[1] Zhou ZB, Huang GX, Fu Q, Han B, Lu JJ, Chen AM (2019). circRNA.33186 contributes to the pathogenesis of osteoarthritis by sponging miR-127-5p. Mol Ther J Am Soc Gene Ther.

[2] Ji ML, Jiang H, Wu F, Geng R, Ya LK, Lin YC (2021). Precise targeting of miR-141/200c cluster in chondrocytes attenuates osteoarthritis development. Ann Rheum Dis.

[3] Chen L, Yu Y (2020). Exercise and osteoarthritis. Adv Exp Med Biol.

[4] Taruc-Uy RL, Lynch SA (2013). Diagnosis and treatment of osteoarthritis. Prim Care.

[5] Rychel JK (2010). Diagnosis and treatment of osteoarthritis. Top Companion Anim Med.

[6] Tafrihi M, Hasheminasab E (2019). MiRNAs: biology, biogenesis, their web-based tools, and databases. MicroRNA.

[7] Zhou SS, Jin JP, Wang JQ, Zhang ZG, Freedman JH, Zheng Y (2018). miRNAS in cardiovascular diseases: potential biomarkers, therapeutic targets and challenges. Acta Pharmacol Sin.

[8] Giordano L, Porta GD, Peretti GM, Maffulli N (2020). Therapeutic potential of microRNA in tendon injuries. Br Med Bull.

[9] Oliviero A, Della Porta G, Peretti GM, Maffulli N (2019). MicroRNA in osteoarthritis: physiopathology, diagnosis and therapeutic challenge. Br Med Bull.

[10] Gargano G, Oliviero A, Oliva F, Maffulli N (2021). Small interfering RNAs in tendon homeostasis. Br Med Bull.

[11] Gargano G, Oliva F, Oliviero A, Maffulli N (2022). Small interfering RNAs in the management of human rheumatoid arthritis. Br Med Bull.

[12] de Sousa MC, Gjorgjieva M, Dolicka D, Sobolewski C, Foti M (2019). Deciphering miRNAs’ action through miRNA Editing. Int J Mol Sci.

[13] Cao Y, Tang S, Nie X, Zhou Z, Ruan G, Han W (2021). Decreased miR-214-3p activates NF-κB pathway and aggravates osteoarthritis progression. EBioMedicine.

[14] Lai C, Liao B, Peng S, Fang P, Bao N, Zhang L. Synovial fibroblast-miR-214-3p-derived exosomes inhibit inflammation and degeneration of cartilage tissues of osteoarthritis rats. Mol Cell Biochem. 2022. 10.1007/s11010-022-04535-9 PMC9938056 36001206

[15] Zhao S, Mi Y, Zheng B, Wei P, Gu Y, Zhang Z (2022). Highly-metastatic colorectal cancer cell released miR-181a-5p-rich extracellular vesicles promote liver metastasis by activating hepatic stellate cells and remodelling the tumour microenvironment. J Extracell Vesicles.

[16] Xue J, Min Z, Xia Z, Cheng B, Lan B, Zhang F (2018). The hsa-miR-181a-5p reduces oxidation resistance by controlling SECISBP2 in osteoarthritis. BMC Musculoskelet Disord.

[17] Mo J, Liang H, Su C, Li P, Chen J, Zhang B (2021). DDX3X: structure, physiologic functions and cancer. Mol Cancer.

[18] Lennox AL, Hoye ML, Jiang R, Johnson-Kerner BL, Suit LA, Venkataramanan S (2020). Pathogenic DDX3X mutations impair RNA metabolism and neurogenesis during fetal cortical development. Neuron.

[19] Clough E, Barrett T (2016). The gene expression omnibus database. Methods Mol Biol.

[20] Franceschini A, Szklarczyk D, Frankild S, Kuhn M, Simonovic M, Roth A (2013). STRING v9.1: protein–protein interaction networks, with increased coverage and integration. Nucleic Acids Res.

[21] Shannon P, Markiel A, Ozier O, Baliga NS, Wang JT, Ramage D (2003). Cytoscape: a software environment for integrated models of biomolecular interaction networks. Genome Res.

[22] Mon-López D, Tejero-González CM (2019). Validity and reliability of the TargetScan ISSF Pistol & Rifle application for measuring shooting performance. Scand J Med Sci Sports.

[23] Chien SY, Huang CY, Tsai CH, Wang SW, Lin YM, Tang CH (2016). Interleukin-1β induces fibroblast growth factor 2 expression and subsequently promotes endothelial progenitor cell angiogenesis in chondrocytes. Clin Sci.

[24] Abdel-Aziz MA, Ahmed HMS, El-Nekeety AA, Abdel-Wahhab MA (2021). Osteoarthritis complications and the recent therapeutic approaches. Inflammopharmacology.

[25] Binvignat M, Sokol H, Mariotti-Ferrandiz E, Berenbaum F, Sellam J (2021). Osteoarthritis and gut microbiome. Jt Bone Spine.

[26] D’Arcy Y, Mantyh P, Yaksh T, Donevan S, Hall J, Sadrarhami M (2021). Treating osteoarthritis pain: mechanisms of action of acetaminophen, nonsteroidal anti-inflammatory drugs, opioids, and nerve growth factor antibodies. Postgrad Med.

[27] Thorlund JB, Simic M, Pihl K, Berthelsen DB, Day R, Koes B (2022). Similar effects of exercise therapy, nonsteroidal anti-inflammatory drugs, and opioids for knee osteoarthritis pain: a systematic review with network meta-analysis. J Orthop Sports Phys Ther.

[28] Sanchez-Lopez E, Coras R, Torres A, Lane NE, Guma M (2022). Synovial inflammation in osteoarthritis progression. Nat Rev Rheumatol.

[29] Wang C, Wang L, Guan X, Yue C (2021). MiR-4303 relieves chondrocyte inflammation by targeting ASPN in osteoarthritis. J Orthop Surg Res.

[30] Samir P, Kesavardhana S, Patmore DM, Gingras S, Malireddi RKS, Karki R (2019). DDX3X acts as a live-or-die checkpoint in stressed cells by regulating NLRP3 inflammasome. Nature.

[31] Jin J, Lv X, Wang B, Ren C, Jiang J, Chen H (2021). Limonin inhibits IL-1β-induced inflammation and catabolism in chondrocytes and ameliorates osteoarthritis by activating Nrf2. Oxid Med Cell Longev.

[32] Lee CH, Chiang CF, Kuo FC, Su SC, Huang CL, Liu JS (2021). High-molecular-weight hyaluronic acid inhibits IL-1β-induced synovial inflammation and macrophage polarization through the GRP78-NF-κB signaling pathway. Int J Mol Sci.

[33] Cui SB, Wang TX, Liu ZW, Yan JY, Zhang K (2021). Zinc finger protein A20 regulates the development and progression of osteoarthritis by affecting the activity of NF-κB p65. Immunopharmacol Immunotoxicol.

[34] Hu L, Luo D, Zhang H, He L (2022). Polydatin inhibits IL-1β-mediated chondrocyte inflammation and ameliorates cartilage degradation: Involvement of the NF-κB and Wnt/β-catenin pathways. Tissue Cell.

[35] Zhang X, Hsueh MF, Huebner JL, Kraus VB (2021). TNF-α carried by plasma extracellular vesicles predicts knee osteoarthritis progression. Front Immunol.

[36] Chiu YS, Bamodu OA, Fong IH, Lee WH, Lin CC, Lu CH (2021). The JAK inhibitor Tofacitinib inhibits structural damage in osteoarthritis by modulating JAK1/TNF-alpha/IL-6 signaling through Mir-149-5p. Bone.

[37] Lin Z, Liu T, Hu Z, Que W, Qiu H, Chen L (2022). Effects of different running intensity on serum levels of IL-6 and TNF-α in patients with early knee osteoarthritis. J Coll Phys Surg-Pak JCPSP.

[38] Barrera-Rojas CH, Otoni WC, Nogueira FTS (2021). Shaping the root system: the interplay between miRNA regulatory hubs and phytohormones. J Exp Bot.

[39] Lu Y, Wang YL, Liu Q, Zhou P, Mei PY, Li JS (2021). MiRNA-122 promotes ischemia-reperfusion injury after lung transplantation via the toll-like receptor signaling pathway. Curr Med Sci.

[40] Pietrykowska H, Sierocka I, Zielezinski A, Alisha A, Carrasco-Sanchez JC, Jarmolowski A (2022). Biogenesis, conservation, and function of miRNA in liverworts. J Exp Bot.

[41] Li S, He Y, Chen K, Sun J, Zhang L, He Y (2021). RSL3 drives ferroptosis through NF-κB pathway activation and GPX4 depletion in glioblastoma. Oxid Med Cell Longev.

[42] Volmar MNM, Cheng J, Alenezi H, Richter S, Haug A, Hassan Z (2021). Cannabidiol converts NF-κB into a tumor suppressor in glioblastoma with defined antioxidative properties. Neuro Oncol.

[43] Catheline SE, Bell RD, Oluoch LS, James MN, Escalera-Rivera K, Maynard RD (2021). IKKβ-NF-κB signaling in adult chondrocytes promotes the onset of age-related osteoarthritis in mice. Sci Signal.

[44] Statement of Retraction (2022). The protective role of microRNA-140-5p in synovial injury of rats with knee osteoarthritis via inactivating the TLR4/Myd88/NF-κB signaling pathway. Cell Cycle.

[45] Fu Y, Lei J, Zhuang Y, Zhang K, Lu D (2016). Overexpression of HMGB1 A-box reduced IL-1β-induced MMP expression and the production of inflammatory mediators in human chondrocytes. Exp Cell Res.

[46] Burrage PS, Mix KS, Brinckerhoff CE (2006). Matrix metalloproteinases: role in arthritis. Front Biosci.

[47] Mehana EE, Khafaga AF, El-Blehi SS (2019). The role of matrix metalloproteinases in osteoarthritis pathogenesis: an updated review. Life Sci.

